# MicroRNA miR-146a-5p inhibits the inflammatory response and injury of airway epithelial cells via targeting TNF receptor-associated factor 6

**DOI:** 10.1080/21655979.2021.1927545

**Published:** 2021-05-18

**Authors:** Fang Yan, Dilinuer Wufuer, Jianbing Ding, Jing Wang

**Affiliations:** a Department of Respiratory Medicine, The First Affiliated Hospital of Xinjiang Medical University, Urumqi, Xinjiang, P.R. China; b School of Public Health, Xinjiang Medical University, Urumqi, Xinjiang, P.R. China; c Department of Immunology, College of Basic Medicine, Xinjiang Medical University, Urumqi, Xinjiang, P.R. China; d Department of Respiratory Medicine, The Second Affiliated Hospital of Hainan Medical University, Haikou, Hainan, P.R. China.

**Keywords:** Asthma, miR-146a-5p, traf6, inflammation, cell barrier injury, apoptosis

## Abstract

Bronchial asthma is a common respiratory disease, which is characterized by airway inflammation, remodeling and hyperresponsiveness. MicroRNAs (miRNAs), as reported, are implicated in the pathogenesis of many diseases, but how miRNAs-146a-5p (miR-146a-5p) works in asthma remains inconclusive. In this work, we proved that miR-146a-5p expression was inhibited in asthma patients’ plasma and platelet activating factor (PAF)-induced human small airway epithelial cells (HSAECs). MiR-146a-5p up-regulation ameliorated the inflammatory reaction and cell barrier damage of HSAECs induced by PAF, and inhibited the apoptosis; besides, miR-146a-5p down-regulation functioned oppositely. In addition, miR-146a-5p could target TNF receptor-associated factor 6 (TRAF6) and negatively regulate its expression. TRAF6 overexpression could counterract the impact of miR-146a-5p up-regulation on PAF-induced inflammation, cell barrier damage and apoptosis of HSAECs. Collectively, miR-146a-5p may protect airway epithelial cells and inhibit the pathogenesis of asthma via targeting TRAF6.

## Graphical abstract

**Figure:**
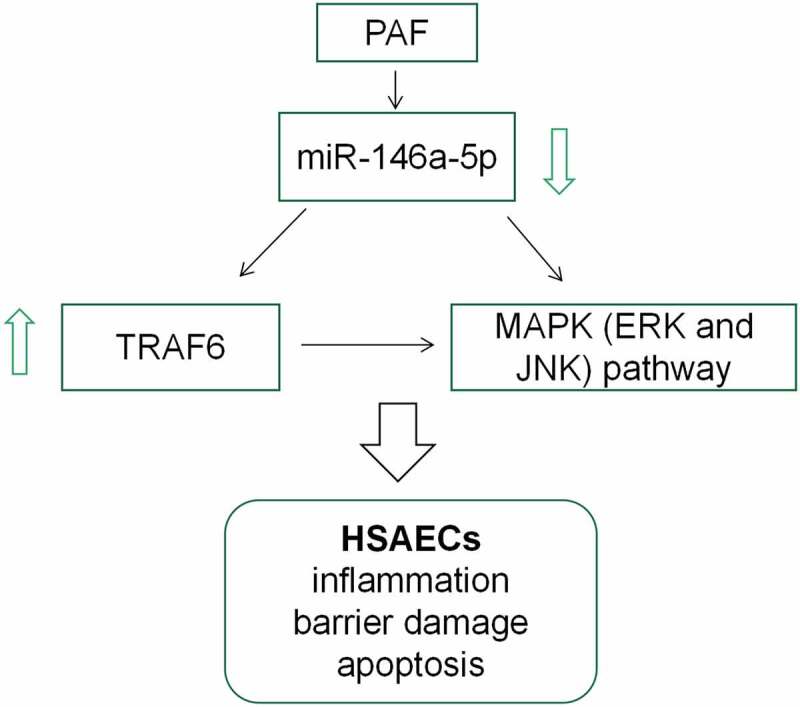

## Introduction

Asthma is a common chronic respiratory disease impacting about 300 million patients in the globe [1,2]. Asthma is associated with airway inflammatory reaction, which is mediated by a variety of inflammatory cells and mediators [3]. Among them, airway epithelial cells, the barrier against external environment, are vulnerable in airway inflammation [4]. Corticosteroids and long-acting beta agonists alleviate the symptoms of the patients, but for some patients, their effects are not satisfactory [5,6]. It is indispensable to explore the pathogenesis of asthma and find new strategies to counteract the airway inflammation and reduce the airway epithelial injury.

MicroRNA (miRNA), known as non-coding small RNA with about 19 ~ 25*nt* in length, plays a regulatory role in various biological activities at the post-transcriptional level [7–9]. MiRNA, as reported, is vital in modulating airway inflammation in asthma [10,11]. For example, miR-181b-5p is lowly expressed in airway epithelial tissue and plasma of asthma patients, and it can inhibit the expression of proinflammatory cytokines by targeting SPP1, thus participating in the inhibition of eosinophilic airway inflammation [10]; miR-155 improves the viability of Th cells by down-regulating CTLA-4 expressions, thus inducing asthma [11]. Reportedly, miR-146a-5p is declined in allergic asthma in mice, and its up-regulation can reduce the activities of COX-2 and 5-LO [12]. Instead, how miR-146a-5p functions in asthma has not been thoroughly clarified.

TNF receptor-associated factor 6 (TRAF6), belonging to the TRAF family, is a transducer for inflammatory signaling [13,14]. TRAF6 regulates thymic epithelial cell differentiation, bone metabolism, innate immune response, dendritic cell maturation and embryonic development [15,16]. TRAF6 mRNA is highly expressed in the trachea of obese asthmatic male Wistar rats [17]. It is reported that miR-146a restrains the secretion of IL-33 in type II innate lymphoid cells by blocking IRAK1 and TRAF6 expression, thus inhibiting airway inflammation in asthma [18].

This study was designed to explore miR-146a-5p expression characteristics in asthma and its biological functions in airway inflammation and epithelial barrier damage with *in-vitro* models. We report that miR-146a-5p is implicated in regulating airway inflammation and the injury of airway epithelial cells in asthma via targeting TRAF6.

## Materials and methods

### Clinical samples

Blood samples of 57 patients with acute asthma, treated in the First Affiliated Hospital of Xinjiang Medical University from June 2018 to August 2019 were collected, with 57 cases of blood samples of healthy volunteers as negative controls. Samples are stored at −80°C immediately after collection. The research scheme, with all subjects’ informed consents, was endorsed by the Ethics Review Committee of the First Affiliated Hospital of Xinjiang Medical University (Approval Number: 2,018,002).

### Cell culture

Human small airway epithelial cells (HSAECs), available from American Type Culture Collection (ATCC; Manassas, VA, USA), were cultured in Dulbecco Modified Eagle Medium (Invitrogen, Carlsbad, CA, USA) with 10% fetal bovine serum (FBS) and 1% penicillin/streptomycin (Hyclone, Logan, UT, USA) at 37°C in 5% CO_2_. Platelet activating factor (PAF) was available from Merck (Darmstadt, Germany) and the working PAF solution was accordingly prepared by dimethylsulfoxide (DMSO) (MackLin Biochemical Technology Co., Ltd., Shanghai, China). HSAECs in logarithmic phase were stimulated by PAF to construct asthma cell model.

### Cell transfection

MiR-146a-5p mimics (miR-146a-5p), mimics negative control (miR-NC), miR-146a-5p inhibitors (miR-146a-5p-in), inhibitors negative control (miR-in), empty plasmid and TRAF6 overexpression plasmid were available from GenePharma (Shanghai, China). PAF-induced HSAECs were transfected by lipofectamine^TM^ 3000 (Invitrogen, Carlsbad, CA, USA), and 48 h later, the transfection efficacy was accordingly examined by quantitative real-time polymerase-chain reaction (qRT-PCR).

### qRT-PCR

Total RNA was subsequently extracted from plasma of asthma patients and HSAECs by Trizol reagent (Invitrogen, Waltham, MA, USA). Besides, the miRNA and mRNA were reversely transcribed into cDNA by PrimeScript RT Master Mix (TaKaRa, Dalian, China). For the reverse transcription of miR-146a-5p, stem-loop method was used, and the sequence of the primer is: 5'-GTCGTATCCAGTGCAGGGTCCGAGGTATTCGCACTGGATACGACAACCCAT-3'. qRT-PCR was performed on ABI 7900 system (Applied Biosystems, Carlsbad, CA, USA) with SYBR® Select Master Mix (Roche, Shanghai, China). The relative expressions were calculated by 2^−ΔΔCt^ method, with GAPDH and U6 as the endogenous controls. Primer sequences used in this work: miR-146a-5p: forward: 5'-CGCGTGAGAACTGAATTCCA-3'; reverse: 5'-GTCGTATCCAGTGCAGGG-3'. TRAF6: forward: 5'-CTCAGCGCTGTGCAAACTATATATCCC-3'; reverse: 5'-GGCGTATTGTACCCTGGAAGGG-3'. IL-6: forward:5'-GTGAAAGCAGCAAAGAGGC-3'; reverse: 5'-CATTTGTGGTTGGGTCAGG-3'. IL-1β: forward:5'-TACGAATCTCCGACCACCACTACAG-3'; reverse: 5'-TGGAGGTGGAGAGCTTTCAGTTCATATG-3'. TNF-α: forward: 5'-CTCCACCCATGTGCTCCTCAC-3'; reverse: 5'-CCCAAAGTAGACCTGCCCAGA-3'. U6: forward: 5'-CTCGCTTCGGCAGCACA-3'; reverse: 5'-AACGCTTCACGAATTTGCGT-3'. GAPDH: forward: 5'-GAAGGTGAAGGTCGGAGT-3'; reverse: 5'-GAAGATGGTGATGGGATTTC-3'.

### Enzyme-linked immune sorbent assay (ELISA)

ELISA kits (TaKaRa, Dalian, China) were adopted to examine interleukin-1β (IL-1β), interleukin-6 (IL-6) and tumor necrosis factor (TNF-α) levels. Cells were cultured in a 6-well plate at 37°C in 5% CO_2_ for 24 h and starved for 12 h. Besides, the cell supernatant was subsequently collected, with the concentration of inflammatory cytokines detected according to the manufacturer’s instructions.

### Epithelial monolayer permeability assay

HSAECs were inoculated on the upper surface of the microporous membrane of Transwell chamber at 1 × 10^5^ cells/cm^2^ (diameter: 6.5 mm, pore size: 0.4 μ m; Corning incorporated, Corning, NY, USA), and the experiment started after the cells reached 100% confluence and formed a dense monolayer barrier. The cells were rinsed carefully with Hank’s Balanced Salt Solution (HBSS) (pH 7.4) for 3 times, and incubated at 37°C for 30 min, and then the HBSS in the wells was removed. Then, 80 mg/L Lucifer Yellow (Sigma-Aldrich, Louis, MO, USA) was loaded into the upper compartment of the Transwell chambers, and the cells were cultivated at 37°C for 1 h. Next, the fluids in the below compartments were collected. At last, the fluorescence (excitation wavelength: 427 nm, emission wavelength: 536 nm) was detected by a microplate reader (BioTek, Winooski, VT, USA).

### Western blot

Cells in different groups were, respectively, lysed with RIPA lysis buffer (Beyotime, Shanghai, China), and the supernatant was subsequently collected followed by centrifugation to extract the total protein, with its concentration probed by a BCA protein assay kit (Solarbio, Beijing, China). Notably, equal amount (20 μg) of protein samples in each group were separated by SDS-PAGE, and transferred to polyvinylidene fluoride membrane (Millipore, Bedford, MA, USA), which was blocked with 5% skimmed milk for 1 h at ambient temperature, and specifically incubated with primary antibody at 4°C overnight. TRAF6 antibody (ab33915, 1:1000) and GAPDH antibody (ab181602, 1:1000) were purchased from Abcam (Shanghai, China). phospho-JNK antibody (p-JNK; #4668, 1:1000), phospho-ERK antibody (p-ERK; #4370, 1:1000), JNK antibody (#9252, 1:1000) and ERK1/2 antibody (# 4695, 1:1000) were available from Cell Signaling Technology (Danvers, MA, USA). Afterward, the membranes were incubated with goat anti-rabbit IgG (ab205718, 1:3000, Abcam, Shanghai, China) for 1 h at 37°C. Ultimately, the protein bands were developed by enhanced chemiluminescence kit (Solarbio, Beijing, China).

### Flow cytometry analysis

Briefly, the transfected cells were collected, rinsed twice in pre-cooled PBS, and accordingly resuspended in 400 μL of binding buffer (Sungene Biotech, Tianjin, China). Equal volume (5 μL) of fluorescein isothiocyanate (FITC)-labeled Annexin V solution and propidium iodide (PI) staining solution (Sungene Biotech, Tianjin, China) were mixed, added into the cell suspension and incubated in the dark at ambient temperature for 15 min. Then, the apoptosis of the HSAECs was examined by a flow cytometer (BD Biosciences, San Jose, CA, USA).

### Dual-luciferase reporter gene assay

The wild type (WT) or mutant (MUT) of TRAF6 3'-UTR was amplified and cloned into psiCHECK-2 vector (Promega, Madison, WI, USA) to establish luciferase reporter vectors (TRAF6-WT or TRAF6-MUT). TRAF6-WT or TRAF6-MUT was then co-transfected with miR-146a-5p or miR-NC into HSAECs. 48 h later, the luciferase activity was assessed by dual-luciferase reporter kit (Promega, Madison, WI, USA).

### Statistical analysis

All assays in the present work were conducted in triplicate, and the data were expressed as ‘mean ± standard deviation (SD)’. The data between two groups was compared by student’s *t* test. One-way ANOVA and Tukey’s post hoc test were utilized to measure the differences among multiple groups. Pearson’s correlation analysis was utilized to study the correlations between miR-146a-5p and TRAF6 mRNA expressions. Statistically, *P* < 0.05 is meaningful.

## Results

This work was aimed to probe miR-146a-5p expression characteristics in the plasma of asthma patients and PAF-induced HSAECs, and to study the effects of miR-146a-5p on PAF-induced inflammatory response, cell barrier damage and apoptosis of HSAECs as well as the underlying mechanism. This work demonstrated that miR-146a-5p expression was reduced in asthma patients’ plasma and PAF-induced HSAECs. MiR-146a-5p up-regulation reduced the inflammatory reaction and cell barrier damage of HSAECs induced by PAF, and inhibited the apoptosis, while miR-146a-5p inihibition functioned oppositely. In addition, TRAF6 overexpression counterbalanced the impacts of miR-146a-5p up-regulation on PAF-induced inflammation, cell barrier damage and HSAECs apoptosis. Overall, this study shows that miR-146a-5p inhibits the pathogenesis of asthma by targeting TRAF6.

### MiR-146a-5p expression level is reduced in the plasma of HSAEC of patients with asthma and PAF-stimulated HSAECs

**Figure 1.**
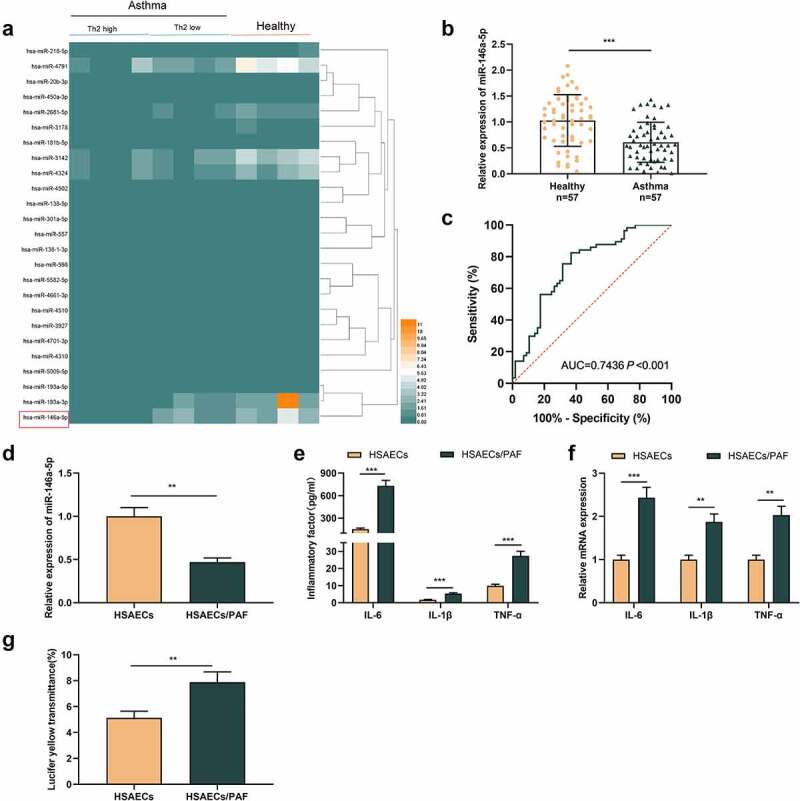
MiR-146a-5p is lowly expressed in the blood samples and HSAECs treated with PAFA

First of all, we analyzed the microarray data from GSE142237, which included the miRNA expression profile of bronchial epithelial brushings obtained by bronchoscopy from eight asthmatics and four healthy controls. It was observed that miR-146a-5p expression level was lower in asthma group than that in the control group (Figure 1a). We also detected miR-146a-5p expression in the plasma of 57 patients and 57 healthy subjects by qRT-PCR, and consistently, it was revealed that miR-146a-5p expressions in the plasma of asthmatic patients were lower (Figure 1b). ROC analysis highlighted that miR-146a-5p had promising diagnostic accuracy for asthma (area under the ROC curve [AUC] = 0.7436; *P* <0.001) (Figure 1c). Then, HSAECs was treated with PAF, which could induce the inflammatory response. As against HSAECs in the control, miR-146a-5p expression was declined in HSAECs/PAF group (Figure 1d). ELISA indicated that compared with HSAECs in the control group, inflammatory cytokines’ contents in HSAECs/PAF group were up-regulated (Figure 1e). qRT-PCR highlighted that the mRNA expressions of inflammatory cytokines in HSAECs/PAF group were also up-regulated (figure 1f). In addition, we used fluorescence yellow transmittance as an index to analyze the changes of permeability of HSAECs treated with PAF. As shown, the fluorescence yellow transmittance of HSAECs/PAF group was demonstrably higher than that of HSAECs in the control group, suggesting that PAF could probably induce the injury of epithelial barrier (Figure 1g).

(A) Heat map showed the difference of miRNA expression profile between the bronchial epithelium of asthma patients and healthy volunteers in GSE142237. (B) qRT-PCR was adopted to detect the expression of miR-146a-5p in the plasma of healthy volunteers and asthma patients. (C) ROC curve was plotted to evaluate the diagnostic value of plasma miR-146a-5p in asthma. (D) qRT-PCR was adopted to detect the expression of miR-146a-5p in HSAECs with or without PAF stimulation. (E) ELISA was used to detect the levels of IL-1β, IL-6 and TNF-α in the supernatnat of HSAECs treated with PAF. (F) The mRNA expression of IL-1β, IL-6 and TNF-α in HSAECs treated with PAF was detected by qRT-PCR. (G) With the fluorescence yellow transmittance as an index, the permeability of HSAECs was detected by Transwell assay. All experiments were performed in triplicate. **P < 0.01, and ***P < 0.001.

### Influences of miR-146a-5p on PAF-caused inflammation response and injury of HSACEs

**Figure 2.**
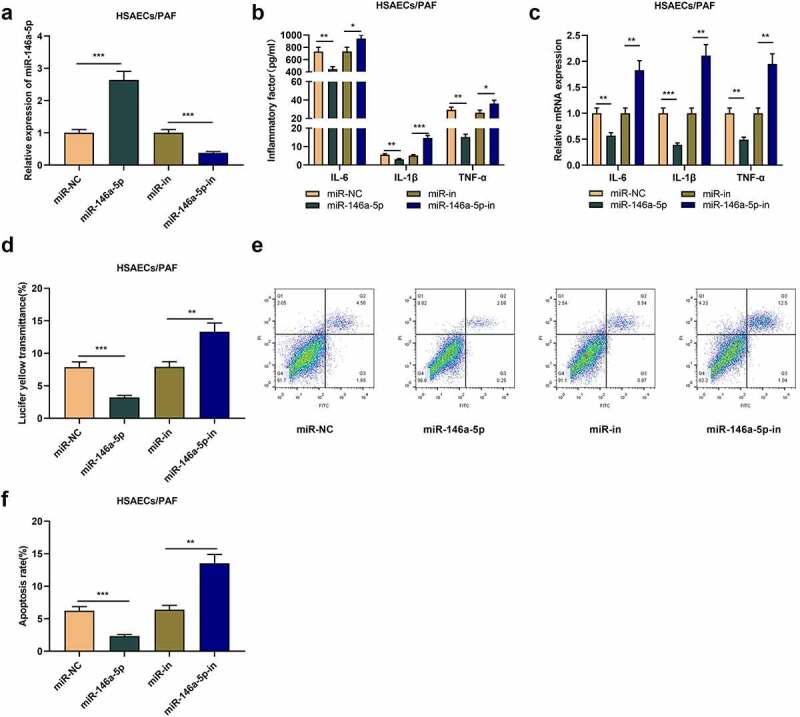
The effect of miR-146a-5p on airway inflammation and epithelial cell barrier injury in asthma

To study the role of miR-146a-5p in asthma, we transfected miR-146a-5p mimics or inhibitors into HSAECs treated with PAF (Figure 2a). As a result, ELISA and qRT-PCR uncovered that relative to the control group (miR-NC or miR-in), miR-146a-5p up-regulation greatly decreased the contents of inflammatory cytokines in HSAECs, while inhibiting miR-146a-5p worked oppositely (Figure 2b-c). What is more, miR-146a-5p overexpression restrained the fluorescent yellow transmittance of HSAECs, while miR-146a-5p inhibition worked oppositely (Figure 2d). Flow cytometry analysis proved that miR-146a-5p overexpression inhibited the apoptosis of HSAECs, while the apoptosis rate of HSAECs was increased upon transfecting miR-146a-5p inhibitors (Figure 2e-f). Collectively, miR-146a-5p could restrain PAF-induced inflammation response of HSACEs and reduce the damage of cell barrier in asthma.

(A) The mRNA expression miR-146a-5p in PAF-treated HSAECs transfected with miR-146a-5p mimics or inhibitors was detected by qRT-PCR. (B) IL-1β, IL-6 and TNF-α levels in the supernatnat of PAF-treated HSAECs transfected with miR-146a-5p mimics or inhibitors were detected by ELISA. (C) The mRNA expressions of IL-1β, IL-6 and TNF-α in PAF-treated HSAECs transfected with miR-146a-5p mimics or inhibitors were detected by qRT-PCR. (D) The permeability of PAF-treated HSAECs transfected with miR-146a-5p mimics or inhibitors was detected by Transwell assay. (E) and (F) Flow cytometry was used to detect the apoptosis of PAF-treated HSAECs transfected with miR-146a-5p mimics or inhibitors. All experiments were performed in triplicate. *P < 0.05, **P < 0.01, and ***P < 0.001.

### MiR-146a-5p directly targets TRAF6

**Figure 3.**
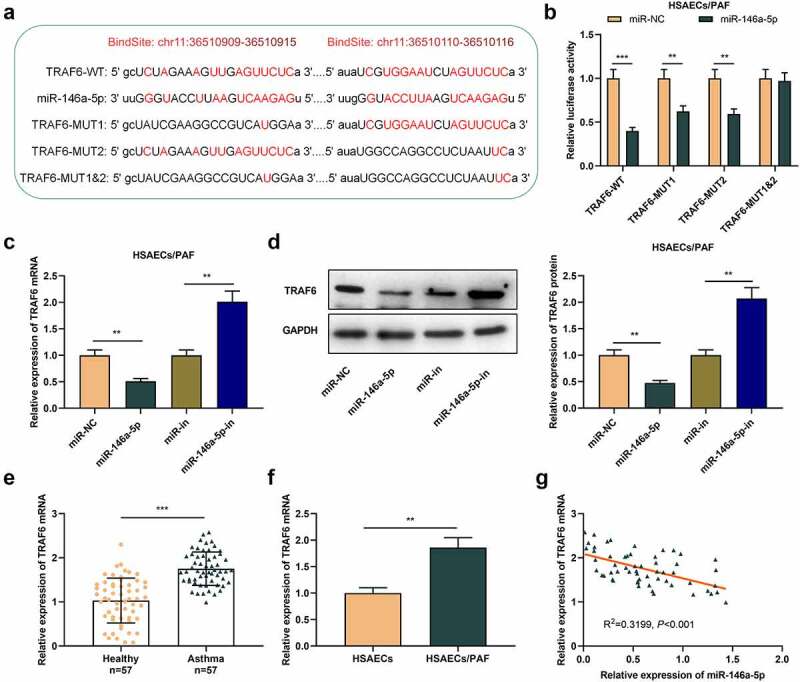
TRAF6 is the downstream target of miR-146a-5p in HSAECs

To delve into the potential mechanism of miR-146a-5p in asthma, we predicted the potential downstream targets of miR-146a-5p by StarBase database and observed that there was a complementary binding site between miR-146a-5p and TRAF6 mRNA 3'UTR (Figure 3a). Subsequently, dual-luciferase reporter gene assay suggested that miR-146a-5p up-regulation restrained the luciferase activity of TRAF6-WT, TRAF6-MUT1 and TRAF6-MUT2 reporters, but that of TRAF6-MUT1&2 reporter was unaffected (Figure 3b). qRT-PCR and western blot uncovered that TRAF6 mRNA and protein levels in asthma cells transfected with miR-146a-5p mimics were greatly declined, and transfection of miR-146a-5p inhibitors functioned oppositely (Figure 3c-d). Collectively, TRAF6 was a target of miR-146a-5p in HSACEs.

(A) Bioinformatics analysis predicted the binding sequence between miR-146a-5p and TRAF6 3'-UTR. (B) Dual-luciferase reporter gene assay was used to verify the targeting relationship between miR-146a-5p and TRAF6. (C) and (D) qRT-PCR and western blot were used to detect the expression of TRAF6 mRNA and the protein level in PAF-treated HSAECs transfected with miR-146a-5p mimics or inhibitors. All experiments were performed in triplicate. **P < 0.01, and ***P < 0.001.

### TRAF6 overexpression counterbalances the impact of up-regulated miR-146a-5p on inflammatory response of HSAECs induced by PAF

**Figure 4.**
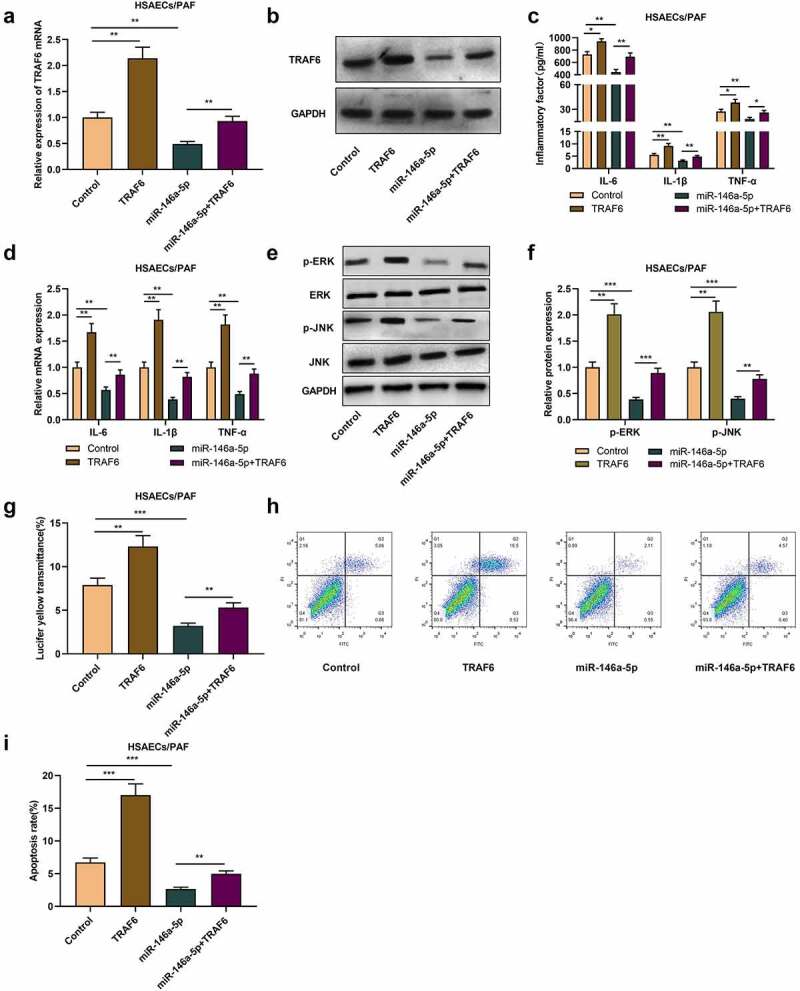
MiR-146a-5p regulates the inflammation and injury of HSAECs by targeting TRAF6

Subsequently, we co-transfected TRAF6 overexpression plasmid into HSAECs transfected with miR-146a-5p mimics. qRT-PCR and western blot uncovered that as against the control group, overexpression of TRAF6 increased TRAF6 mRNA and protein levels in HSAECs, and reversed the decrease of TRAF6 expression caused by miR-146a-5p up-regulation (Figure 4a-b). ELISA and qRT-PCR proved that compared with control group, the levels of inflammatory cytokines in cells with TRAF6 overexpression were significantly increased, meanwhile, overexpression of TRAF6 could also weaken the impact of miR-146a-5p mimics on inflammatory cytokines levels (Figure 4c-d). Besides, western blot proved that transfecting miR-146a-5p mimics suppressed p-ERK and p-JNK protein levels, while overexpression of TRAF6 functioned oppositely (Figure 4e-f). Additionally, relative to the control group, the fluorescent yellow transmittance of the cells in TRAF6 overexpression group was dramatically enhanced, and compared with the cells in miR-146a-5p overexpression group that of miR-146a-5p+TARF6 group were also enhanced (Figure 4g). Flow cytometry proved that overexpression of TRAF6 facilitated PAF-induced apoptosis and weakened the impact of overexpression of miR-146a-5p on apoptosis (Figure 4h-i), indicating that miR-146a-5p can reduce airway inflammation and cell barrier damage in asthma by targeting TRAF6.

(A) and (B) qRT-PCR and western blot were used to detect the transfection efficiency of miR-146a-5p mimics and TRAF6 overexpression plasmids in PAF-treated HSAECs. (C) and (D) After transfection, ELISA and qRT-PCR were used to detect the expression of IL-1β, IL-6 and TNF-α in PAF-treated HSAECs. (E) and (F) The protein levels of p-ERK, p-JNK, ERK and JNK in PAF-treated HSAECs were detected by Western blot. (G) Transwell assay was used to detect the permeability of PAF-treated HSAECs. (H) and (I) The apoptosis rate of PAF-treated HSAECs was analyzed by flow cytometry. All experiments were performed in triplicate. *P < 0.05, **P < 0.01, and ***P < 0.001.

## Discussion

Asthma is a chronic inflammatory disease of the airway [3,19]. It is worth noting that airway inflammation is one of the main pathological changes in asthma, which is characterized by the infiltration of mast cells, neutrophils, Th2 cells and eosinophils, and accompanied by the excessive production of inflammatory factors [20]. Airway epithelial cells are a kind of airway structural cells, which function as physical barriers, and they also play immunomodulatory roles by secreting a variety of bioactive substances [4,19,21]. In addition, the progression of asthma inflammation is also associated with a variety of inflammatory mediators, including PAF [22–24]. In this study, we found that PAF stimulation enhanced inflammatory cytokines levels in HSAECs. PAF, as reported, can enhance the paracellular permeability of epithelial barrier [25]. Therefore, inhibiting PAF-induced inflammatory reaction and epithelial barrier injury are beneficial for relieving asthma symptoms.

Many studies have found that miRNA is crucial in cell differentiation, proliferation and apoptosis, and is related to many human diseases, such as cancer, chronic obstructive pulmonary disease, and interstitial lung disease, and asthma [26–31]. MiR-19a expression is dramatically raised in T lymphocytes infiltrated in the airway of asthma, and miR-19a can regulate PTEN, SOCS1 and A20 to cooperatively induce the production of Th2 cytokines [29]; miR-142-3p takes part in regulating the multiplication and differentiation of airway smooth muscle cells in asthma by inhibiting WNT signaling pathway [30]; miR-149 overexpression can inhibit inflammatory cytokines’ expressions in HSAECs induced by PAF, and alleviate the barrier damage of epithelial cells [31]. Here we reported that miR-146a-5p expression was declined in the blood samples of asthma individuals and PAF-stimulated HSAECs; miR-146a-5p overexpression can inhibit the secretion of inflammatory cytokines in HSAECs treated by PAF, alleviate the barrier damage of epithelial cells, and effectively inhibit the apoptosis. The abovementioned data implied that miR-146a-5p participated in the pathogenesis of asthma, and could probably be a therapy target to ameliorate airway inflammation and epithelial barrier injury.

TRAF6, an ubiquitin ligase, contains a highly conserved C-terminal TRAF domain and a coiled-coil N-terminal activation domain [32]. TRAF6 is a pivotal linker protein in intracellular signal transduction, which can directly or indirectly bind to tumor necrosis factor receptor superfamily or interleukin-1/Toll-like receptor superfamily members; besides, TRAF6 regulates cellular proliferation, differentiation and apoptosis through the activation of NF-κB, JNK/p38, PI3K/AKT and AP-1 pathways, and it also regulates innate and acquired immunity, oxidative stress and inflammation [33–36]. For example, miR-146a-5p can inhibit the secretion of IL-6 and IL-1β in OCCM-30 cells through regulating IRAK1/TRAF6 pathway [35]; resveratrol inhibits lipopolysaccharide-induced inflammatory injury of mouse microglia via modulating miR-146a-5p/TRAF6/NF-κB axis [36]. Here we confirmed that miR-146a-5p could target and negatively modulate TRAF6 expressions in HSAECs. Notably, it is reported that the stimulation of PAF can activate MAPK (ERK and JNK) pathway, and thus aggravate the asthma [25]. In this study, we found that overexpression of TRAF6 could activate the MAPK pathway and counteract the inhibitory effects of miR-146a-5p up-regulation on the activation of MAPK signaling. In addition, the overexpression of TRAF6 aggravated airway inflammation and epithelial barrier injury, and reversed the ameliorative effects of miR-146a-5p. Collectively, we concluded that miR-146a-5p/TRAF6 axis is a crucial mechanism of asthma pathogenesis.

### Conclusion

In conclusion, miR-146a-5p ameliorates the inflammatory response and epithelial barrier injury of HSAECs via targeting TRAF6 and repressing MAPK pathway, by which it can inhibit the development of asthma. This work helps clarify the mechanism of asthma pathogenesis. In the following work, *in-vivo* studies are required to further validate our conclusion.

## Highlights

1. MiR-146a-5p is down-regulated in asthma patients’ plasma and PAF-induced HSAECs.

2. MiR-146a-5p reduces PAF-induced HSAECs’ inflammatory response and cell barrier damage, and inhibits cell apoptosis.

3. MiR-146a-5p protects airway epithelial cells via targeting TRAF6.
